# To Correspondents

**Published:** 1868-01-01

**Authors:** 


To Correspondents.—To insure early insertion of your favors it is necessary that they should be condensed to the last limit of perspicuity. Many articles, containing much of real value, have lingered long in the editorial "pigeon holes," because failing in this essential particular. The readers of the Journal, we are proud to know, are well up in the text books and standard treatises. They do not need these elaborated, re-worded, or even quoted. A little reflection on this point will save a great deal of unnecessary manuscript, and, what is of more importance, be vastly more likely to secure careful reading of the communication. Short and pithy articles are read, whilst long ones are postponed to some rainy Sunday or day of leisure, which rarely occurs to the busy gentlemen who take the Journal.

